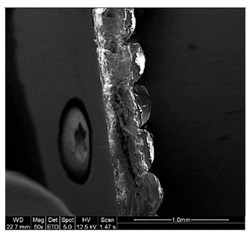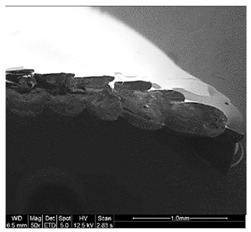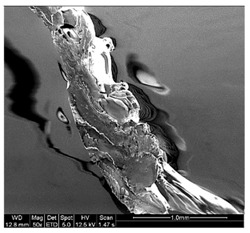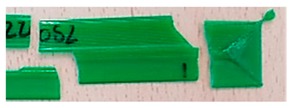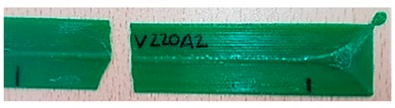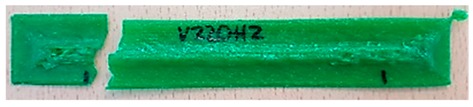# Influence of PLA Filament Conditions on Characteristics of FDM Parts

**DOI:** 10.3390/ma11081322

**Published:** 2018-07-31

**Authors:** Ana Pilar Valerga, Moisés Batista, Jorge Salguero, Frank Girot

**Affiliations:** 1 Department of Mechanical Engineering and Industrial Design, Faculty of Engineering, University of Cadiz, Avenida de la Universidad de Cadiz, 10, Puerto Real, E-11519 Cadiz, Spain; moises.batista@uca.es (M.B.); jorge.salguero@uca.es (J.S.); 2 IKERBASQUE, Basque Foundation for Science, 48013 Bilbao, Spain; frank.girot@ehu.eus; 3 Faculty of Engineering, University of the Basque Country, Alameda de Urquijo s/n, 48013 Bilbao, Spain

**Keywords:** FDM, additive manufacturing, PLA, material color, pigmentation, extrusion temperature, humidity

## Abstract

Additive manufacturing technologies play an important role in Industry 4.0. One of the most prevalent processes is fused deposition modelling (FDM) due to its versatility and low cost. However, there is still a lack of standardization of materials and procedures within this technology. This work aims to study the relationship of certain operating parameters and the conditions of poly(lactic acid) (PLA) polymer with the results of the manufactured parts in dimensional terms, surface quality, and mechanical strength. In this way, the impact of some material characteristics is analyzed, such as the pigmentation of the material and the environmental humidity where it has been stored. The manufacturing parameter that relates to these properties has been the extrusion temperature since it is the most influential in this technology. The results are quite affected especially by humidity, being a parameter little studied in the literature.

## 1. Introduction

Additive manufacturing (AM) has gained increasing acceptance because of issues such as flexibility and design benefits for high value added products. Though these benefits depend on a level of quality regarding material, geometry, and surface finish where significant challenges still remain [1]. However, these technologies already allow the manufacture of a wide range of prototypes as well as functional components with complex and customized geometries, being able to obtain products assembled in a single step and lighten weights through a topology optimization process [2].

Additive manufacturing is at the heart of strategic discussions by the European Factories of the Future Research Association (EFFRA) because of its capabilities in personalized parts, high manufacturing flexibility, and resource efficiency [3].

For this reason, development of this type of technology has experienced a great increase since it was introduced in the market, leading to a set of manufacturing process families that are alternative to subtractive manufacturing or formative manufacturing. However, the need to convert the model into Standard Triangle Language or STL format (mesh of triangles) and the subsequent slicing to obtain the G-code, simplifies the geometry losing resolution in most cases, especially for circular or small parts. This causes improbable geometric deviations, as well as a characteristic roughness associated with layer stacking. Additionally, there is still a lack of information, communication, standardization, and consequent development of these processes [4,5,6].

One of the most widespread processes, due to its versatility and low cost, is fused deposition modelling (FDM) [7,8]. Fused deposition modelling grows almost 20% in profit each year in industries such as automotive or aeronautics [9]. This method generally uses thermoplastic polymers. Despite being one of the most used AM processes, it is not completely industrialized yet. This is due to the large number of parameters that govern the process, as well as the lack of standardization, studies, and communication [9].

Fused deposition modelling is a process that selectively dispenses a thermoplastic polymer through a nozzle. There are specific challenges for this and other additive manufacturing processes, among which are the lack of standardization adapted to each technology and surface quality. In addition, the European Union wants to allow only the consumption of recyclable plastics and eliminate residues of this type of material. For this reason, poly(lactic acid) (PLA) can replace other more used materials, like acrylonitrile butadiene styrene (ABS). Poly(lactic) acid is a natural polymer, derived from renewable sources such as starch, a large carbohydrate that plants synthesize during photosynthesis.

Poly(lactic acid) has played a central role in the replacement of fossil-based polymers for certain applications by being a completely aliphatic polymer.

Furthermore, PLA is a more printable material and has mechanical properties significantly higher than most other plastics except some kinds of polycarbonate, nylon, and composite blends [10,11]. However, this plastic is little studied in relation to the FDM process, despite its great potential because it is renewable, compostable, and biocompatible [12,13,14].

Even though, there are studies that indicate that the working temperature is one of the most important parameters to obtain good quality and accuracy of the PLA parts, by way of other materials [2,15]. Another characteristic barely evaluated is the influence of different pigmentations, whose properties established by the manufacturer are the same regardless of color. This characteristic influences some aspects of the parts according to previous investigations [16].

Likewise, this material is sensitive to humidity, so that environmental conditions may be important in PLA manufacturing. This means that the place where PLA is manufactured has a significant impact on the results, unless the methods are standardized.

Given the importance of the lack of standardization and the influence of the parameters of the material, this paper aims to study some of the final characteristics of the parts obtained with FDM. PLA is used, a biostable material, to take into account the environmental component. In addition, it seeks to increase the component of sustainability without using adhesives for the printing platform, as well as reducing the cost of energy without using heating on the platform. Therefore, the object of this work is the study of the relation of certain studied parameters and environmental non studied parameters with the final quality of the manufactured parts. In this way, the behavior of PLA is analyzed when strategic aspects, such as the environmental humidity of the place where it is stored, the working temperature, and the pigmentation of the material have been modified.

## 2. Materials and Methods

A methodology for studying some manufacturing features is proposed relying on two different tests in order to obtain results that diminish the defects or improve the final characteristics of the manufactured parts by FDM.

**Figure 1:**
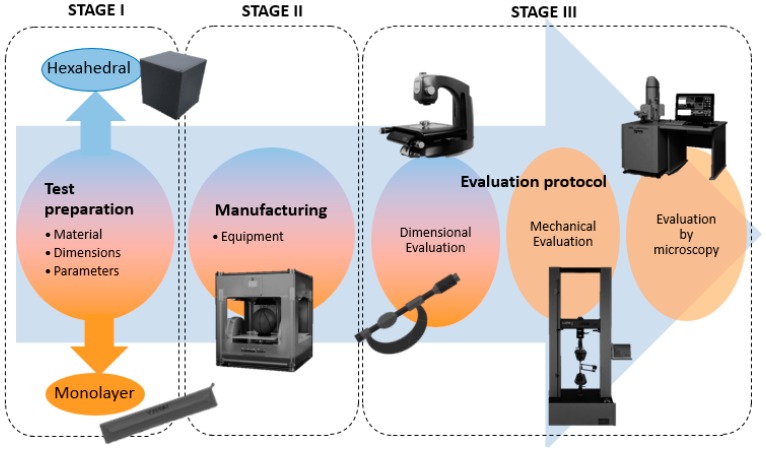
Flow chart of the experimental Procedure.

These two tests have two types of test pieces: hexahedral specimens (30 mm cube) and monolayer specimens. Each trial follows a process divided into three stages, as shown in Figure 1. Hexahedral samples were created in order to measure dimensional deviations more easily and reliably, while monolayers were manufactured for mechanical tests.

On the one hand, hexahedral samples’ infill was 100% rectilinear, 0° raster orientation, with 3 top and bottom layers.

**Figure 2:**
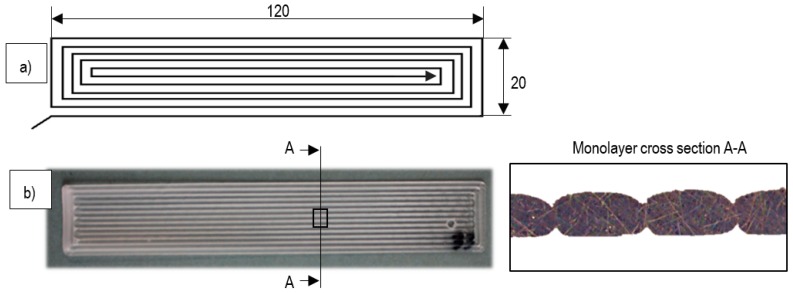
(**a**) Dimensions and trajectories used for monolayer samples [6]; (**b**) monolayer cross-sectional mesostructure.

On the other hand, S-H. Anh et al. [17] have analyzed different possible trajectories for the manufacture of monolayer specimens. Other more recent authors as C. Wendt et al. [6] determined a specific geometry for monolayer samples’ dimensions and trajectories suitable for conducting studies with tensile tests. These dimensions and trajectories are reflected in Figure 2.

Poly (lactic acid), from the same manufacturer BQ, has been used to manufacture the specimens. This material, has properties poorly defined by the manufacturer, Table 1. Specifications include only a few specific ranges. In addition, the manufacturer gives the same characteristics regardless of the selected color, and in this case, nothing is recommended about material storage. However, more adjusted ranges about the characteristics of this material have been obtained in previous studies. Nevertheless, the literature consulted shows different values depending on the manufacturer of the material, and its pigmentation is usually not referred to.

CubeX^®^ Duo was used to manufacture all the samples, highlighting 0.25 mm layer thickness, 30 mm/s feed rate, and an acceleration of 1500 mm/s^2^ as constant parameters. A 1.75 mm filament diameter was printed with a 0.5 mm nozzle size. In addition, 0.5 mm of retraction at 15 mm/s speed was used after ending a path. The print speed was low because no adhesives or heated platform were used. These conditions were also chosen on the basis of previous studies [18]. The study variables of this work are reflected in the Table 2. The Gcode was obtained with the software KISSlicer (1.6.3 version, Trimaker, Buenos Aires, Argentina).

The pigmentation of the filament is an influential factor. For this reason, four different colors (pink, grey, green-transparent, and transparent) have been selected in order to determine if this pigmentation modifies the final properties of the parts. Cubes were manufactured in a temperature range between 180 and 240 °C, but for the monolayer specimens, it was between 200 and 240 °C, due to their difficulty of adhesion to the build platform, owing to the non-use of adhesives and a larger fixing surface than in manufacturing of the cubes (warping).

In relation to the cubic samples, the influence of this pigmentation and the extrusion temperature in the dimensional tolerances and surface quality of the parts were studied. The hexahedral geometry was chosen, since it has all its edges and faces equal, leaving the specimen with a single nominal dimension, which facilitates its analysis.

In order to study the influence of the relative humidity on the results of the monolayer samples, three extreme relative humidity values were used: dry, which corresponds with a relative humidity of 16%; environmental humidity, which corresponds with a relative humidity of 50%; and humid environment, which corresponds with a relative humidity of 98%. This humidity control was achieved by ARL-0680 ESPEC climatic chamber (ESPEC, Osaka, Japan). The temperature of the chamber remained constant at 25 °C. The material was stored in the climate chamber under the conditions defined during a week prior to the manufacturing. Additionally, cubes were manufactured only with material which was stocked in environmental humidity.

Both types of test specimens were subjected to a dimensional evaluation, as well as to an evaluation of the surface quality in terms of roughness average (Ra). The dimensional study was done in terms of deviations from the nominal value (Δd) of each of the main dimensions, where:

(1) $$\Delta d=\bar{X}(\Delta x+\Delta y+\Delta z)$$

The difference between the two tests is that for the hexahedral specimens, the evaluation procedure collected data that was later analyzed.

Additionally, deposited cross-sections of filaments extruded at different temperatures were analyzed to establish the correlation between dimensional deviation and working temperature.

Conversely, the dimensional evaluation of the monolayer specimens serves as the basis for the evaluation of the mechanical properties of the material. These dimensional deviations were characterized using Optical Measures 3D Techniques, concretely Tesa Visio 300 (Tesa SE, Norderstedt, Germany) was used. Also, a contact profilometer, Mahr Perthometer PGK 120 (Mahr GmbH, Göttingen, Germany), was used for microgeometric characterization.

The universal testing machine Shimadzu^®^ AG-X (Shimadzu, Kyoto, Japan) was used for mechanical tests. These tensile tests were executed with a continuous testing velocity of 1 mm/s as recommended in standardization for molding and extrusion plastics [19]. These tests were carried out under conditions of temperature and humidity similar to those of service parts (25 °C and 50%).

Finally, all of the specimens were analyzed before and after the mechanical tests mentioned, to assess by means of stereoscopic optical microscopy (SOM) and scanning electron microscopy (SEM) and support the study of the fracture type that appears in these samples according to their characteristic pigmentations and storage conditions. The equipment used for the application of SOM was Nikon^®^ SMZ800 (Nikon, Chiyoda, Japan), and the EDAX EDS System (Mahwah, NJ, USA) was the equipment used for SEM.

## 3. Results and Discussion

The low homogeneity of the material was verified due to the uncontrolled and arbitrary appearance of the defects typical of this technology. Dissimilar results have been obtained for the same material, under the same starting conditions and with the same path and parameters, which means a low repeatability of the process. This low repetitiveness is associated with the appearance of defects related to the FDM process. Some of them occur frequently and their type and severity are varied. The most outstanding defects analyzed by other authors are: bubbles, cracks, warping, contamination, discolorations, and incorrect binding [6,7].

In spite of this, these can be rectified by modifying some manufacturing parameters. The most complex to solve is the low compaction due to air occlusion (bubbles) or insufficient overlap between filaments that cause cavities.

The appearance of the bubbles will condition the results of both surface quality and tensile strength. The manifestation of this defect, which at lower temperatures is almost non-existent, is due to the fact that at high temperatures the material reaches the extruder nozzle practically liquid. According to other authors, this is due to the fact that at higher temperatures the viscosity decreases [6,7,16]. The PLA, by the time it slides through the nozzle, produces friction with the walls which in turn generates turbulence contributing to the air intake. This results in more bubbles, and therefore in a greater dimensional deviation (Δd), roughness (Ra) and a lower tensile strength.

**Figure 3:**
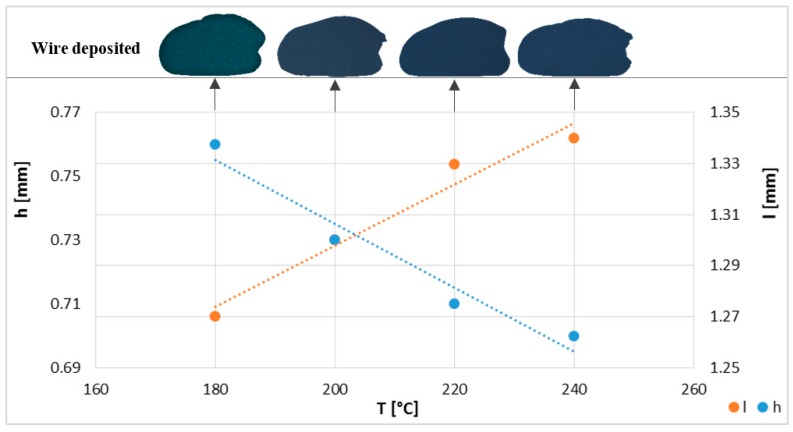
Wire cross-section dimensions of fused deposition modelling (FDM) filament extruded and deposited at different temperatures.

Moreover, by modifying only the extrusion temperature, the deposited section changes not only the dimensions of the specimen but also causes air bubbles between layers. The dimensional values in height (h) and width (l) of extruded filaments deposited at different temperatures are shown in Figure 3. The total Δd can be related to the deviations observed in the section of the filament, since in the final length of the deposited filament (3rd dimension), minor deviations related to the kinematics of the machine have been observed exclusively.

**Figure 4:**
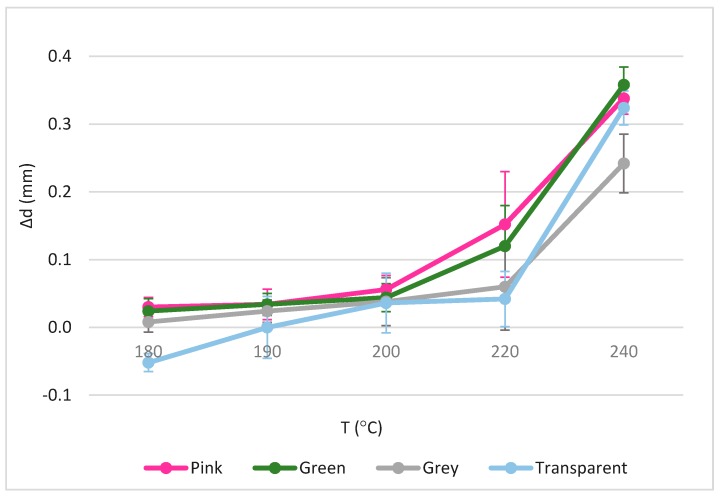
Dimensional deviations in relation to the used temperatures and pigments.

The dimensional determination of the hexahedral specimens was carried out. Figure 4 shows the dimensional deviations (Δd) of the hexahedrons. In both, as the extrusion temperature (T) increases, the pieces also increase in XY dimensions, moving away from the nominal size of 30 mm. On the contrary, the height in the cubes tends to decrease as the manufacturing temperature increases. This is because at higher temperatures the material becomes more fluid, and therefore expands freely by decreasing the measurement on the z-axis.

The different pigments follow the same trend. Even so, some of them differ from the nominal dimension more than others, even having conformed to the same temperature. This proves that the pigmentation has a direct relation with the properties of the parts. Curiously, the lightest colors among those studied, transparent and grey, were kept closer to the nominal measurement than the more intense colors. This preliminary study of the pigments coincides with Aydemir et al. [16].

Moreover, the dimensional deviations were affected by the temperature. Normally, positive deviations are obtained in xy and which increase when the temperature rises, while the layer height decreases, and therefore the height of the complete solid. According to other authors, the material is more fluid and can expand freely in the xy plane [18].

Other material whose pigmentation also lets the light pass is the green test sample. However, this sample behaves very differently than the transparent specimen, so it can be deduced that the green pigment has a very unequal influence on the material even though it has given them the property of being translucent. Surely, if the translucent green material is compared with its opaque counterpart, it would be seen that translucent green behaves much better against dimensional tolerances than opaque [16]. But if compared to other materials with other pigments, whether opaque, translucent or transparent, this color produces parts of worse dimensional quality.

This agrees with other authors who assert that the wettability or the ability to adhere and spread on the surface of a solid is more pronounced in materials with translucent or transparent pigmentation [16]. This favors that the translucent materials stay closer to the nominal measures, because of their ability to adhere to the already solidified layers, reducing the appearance of imperfections. The latter reaffirms the need to standardize each material to be used industrially to avoid that something that is marketed as a material are actually different materials with wide range of properties.

With all this, the dimensional deviations always have little dispersion which are easily solved by redesigning the specimens by establishing a mathematical relationship of the results, or by undergoing a finishing process. However, the mechanical properties do not provide themselves equally to such an advantage.

**Figure 5:**
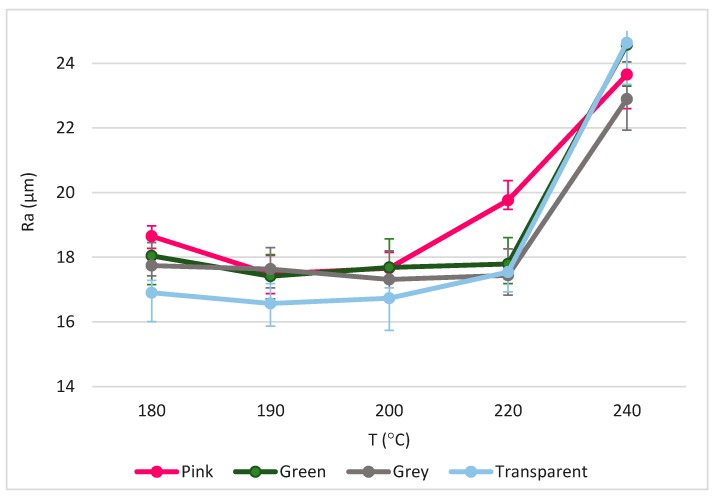
Evolution of roughness average (Ra) as a function of temperature and different pigments.

Nevertheless, surface quality is a major constraint in this manufacturing process [5]. The temperature in the range recommended by the manufacturer apparently does not affect the mechanical properties. Conversely, some pigments can be used in a wider temperature range, as shown in Figure 5. Transparent PLA is usually the material with the lowest value of roughness related to other materials. This is because its viscosity is much greater, adapting the conditions to the formation of a smoother and homogeneous surface, which, according to other authors [16], is caused by its lack of pigment.

Even so, the obtained values of roughness are too high for the vast majority of applications, so that, for implantation in some industries, it would be necessary to use post-processing processes that improve this characteristic of the parts.

The accumulation of defects favored the propagation of cracks in the mechanical tests. It also prevented the test pieces from reaching their elastic limit. This accumulation of defects, mainly bubbles, was aggravated by a higher relative humidity of the environment where the material was stored. Table 3 shows that the storage conditions of the material are even more important than temperature or pigmentation, and it is not often a subject of study [7].

Furthermore, there were some intervals of stresses and deformations of the PLA, regardless of what were their initial conditions, considering between 350 and 900 N the maximum force that it can withstand and between 1 and 4 mm its maximum elongation for all the tests.

Accordingly, the stress–strain curves of the specimens behaved in very similar way. The plastic zone was practically negligible, and the reason why a fragile breakage was predicted. However, since the beginning of the test, small premature fissures occurred throughout the whole piece that made the process irreversible.

In addition, some samples had not been adjusted correctly to the jaws. These measures were rejected in data analyses and corroborated the need to standardize or improve the current standard of the tensile test analysis process for this particular manufacturing process.

In relation to the crack of the samples, this occurred abruptly without strain hardening, indicating that the failure was probably due to the discontinuity in the material and not because it exceeded its limit.

**Figure 6:**
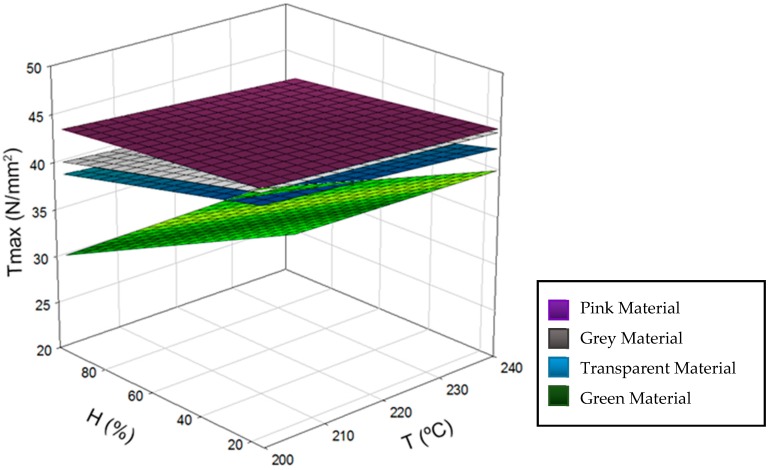
Plot of tensile strength (Tmax) trends as function of variable temperature (T) and relative humidity (H).

Furthermore, comparing the data obtained by families of specimens (Figure 6), it can be seen that specimens whose material was stored in an atmosphere with low relative humidity resisted higher tensile forces. The equations that define the four regression planes corresponding to the tensile strength (Tmax) as a function of the used interval of humidity (H) and temperature (T) coincide with (2–5), (2-grey, 3-pink, 4-transparent, 5-green).

Tmax (T, H) = 3.191 + 0.188 x − 0.048 y(2)

Tmax (T, H) = 3.098 + 0.187 x − 0.011 y(3)

Tmax (T, H) = 3.196 + 0.181 x − 0.048 y(4)

Tmax (T, H) = 3.603 + 0.185 x − 0.171 y(5)

In addition, lower temperature usually created stronger specimens, even if the initial conditions of the material were varied. This is the opposite of what happens with geometric deviations.

If the pigment was prioritized on the mechanical properties of the specimens, it should be noted that those more translucent colors, such as green and transparent, resisted less tensile strength than those characterized by their brightness and opacity.

As in the dimensional and surface quality evaluation, the pigment that gave the worst mechanical properties to the PLA was green again. It is obvious that the pigment seriously modifies the properties of the polymer, making it unsuitable for some applications. However, this should be further explored.

It can be seen that the relative humidity was the most relevant variable studied. This feature is almost never studied in the literature [19]. Specimens whose material was stored in an atmosphere with low relative humidity resisted higher tensile forces. This was due to the degradation that the material undergoes by the action of water. Also, the high temperatures in the nozzle caused water boiling and this transforms into part bubbles. The accumulation of bubbles favored the propagation of cracks in the mechanical tests.

Furthermore, if the elongation of the samples was analyzed, it was observed that the samples made with dry material had less elongation than environmental ones, as shown in Table 4. This resulted in less tenacity or in a more fragile fracture as seen in Table 5.

However, although the transparent color was one of the least resistant, its elongation was greater for the same increase in charge. Thus the absence of pigment returns to the PLA more plastic and malleable, making it able to modify its molecular structure to relieve the internal tensions.

On the other hand, the specimens that were in the dry state, had a very similar value independently of the color and the temperature. In this way, to store the PLA correctly in a cold and dry environment to preserve its mechanical resistance, instead makes it more fragile.

In general, PLA fractures are fragile, an assertion that was true for all specimens tested. However, the breakage varied considerably according to the conditions of the material.

Different sections of specimens made at 220 °C can be seen in Table 5. Although they were manufactured with different initial conditions in the material, it can be seen the discontinuities produced by the degradation of the material or by the appearance of defects linked to humidity.

Material separation, which normally implies severing bonds on the atomic level, is called chain scission. A factor that aids chain scission in PLA is that molecules are not stressed uniformly, because it is an amorphous polymer.

The specimens formed with a material preserved in atmosphere with low relative humidity break through several zones at the same time, so that most of the specimens explode when subjected to the tensile test. This was because the characteristic bubbles were almost non-existent, but the ones that appeared were located in very specific areas, so that the cracks began at the same time in very distributed points. This was due to certain chain segments carrying a disproportionate amount of load when stress was applied, which can be sufficient to exceed the bond strength. The fractures followed a practically straight line, making a clean fracture without detaching small fragments of PLA.

On the other hand, the specimens made with the material stored under environmental conditions, usually broke by a single place, characterized by an uneven fracture. This was because the fissure was propagated by the defects that the material had spread throughout its entire section. Contrary to what happens in dry specimens, small chips of material were released all over the contour of the fracture.

Finally, the specimens made with the material preserved in an atmosphere with high relative humidity had a fracture very similar to the environmental specimens, but this crack was much more pronounced. Therefore, the bubbles had a larger size and they were many more per unit area. In addition, in this case, the crack was no longer always performed by a single place, but several may have appeared along the length of the specimen.

It seems that the oxygen of the water, far from disappearing with increasing temperature of the material, remained in its microstructure causing serious defects that would preclude its use in most industries. In this case, it seems that fracture occurred by chain disentanglement, where molecules separated from one another intact. By increasing the amount of bubbles and subjecting the sample to traction, this defect lengthens until reaching the next closest. This causes that in the breakage there is no distinction between the filaments. In addition, flow lines were observed for the more ductile rupture than in the other cases.

Once again, it is clear the influence of humidity in PLA and the importance of good stocking prior to its manufacture, independently of the parameters chosen for it.

## 4. Conclusions

Additive manufacturing technologies are a new step in the technological revolution towards a 4.0 industry that generates associated services with high added value. This means that there is a need for improvement of these technologies in order to be able to implement them widely in the industry.

One of the necessary steps is the standardization and improvement of the existing regulations as far as materials, processes, and evaluations are concerned. To this end, a methodology was developed to carry out the study of certain characteristics of the material in the results of manufactured parts using FDM technology.

The constant demand for material in this manufacturing process has led to the increase of filament manufacturers, as well as their pigmentation in the market. In this sense, the precise nature of the material will depend on each manufacturer, being unknown, since there are no standards in this regard. For this reason, in each existing study in relation to FDM, the manufacturer is particularized, without specifying in most cases the material color.

In order to approximate this study to the actual industrial context, in this case different specimens have been made from the same base material, from the same manufacturer, stored in different atmospheres, with different pigmentation, and they are extruded at different extrusion temperatures. These three features or characteristics have been related to the results of the parts in terms of dimensional deviations, surface quality, and tensile strength.

On the one hand, it is verified that the materials without pigmentation generally obtain dimensional values closer to the nominal ones. This agrees with studies conducted by other authors that use different manufacturers of filament. In addition, by increasing the extrusion temperature, the material becomes more fluid, which causes an increase in its fluidity and consequently larger dimensional deviations. However, at higher temperatures, up to a maximum of 220 °C, better mechanical strength is achieved.

On the other hand, the lack of pigmentation translates as a greater viscosity of the material, which brings better surface quality to the parts. However, the surface quality of the parts obtained by this technology is often insufficient for most industries, so it would be necessary to develop a post-processing stage.

Finally, PLA is a polymer highly influenced by the environmental conditions in which it is stored. This means that the relative humidity where the material is prior to its manufacture changes its structure. In dry atmospheric conditions, the PLA becomes stronger but less tenacious. A more fragile break is observed, the drier the storage environment.

However, the breakage occurs due to the appearance of defects and not the limit of the material. For this reason, it is essential to achieve a control of the large number of parameters that govern the process, starting with new proposals for standardization of both the material, composition, and storage, as well as the procedures for manufacturing and analyzing the parts.

## Figures and Tables

**Table 1:** Characteristics of the poly(lactic acid) (PLA) according to the manufacturer.

Property	Value
Tensile Strength	16–114 MPa
Elongation at Break	0.5–430%
Modulus of Elasticity	0.230–13.8 GPa
Melting Point	120–170 °C
Working Temperature	180–200 °C
Softening Point	45–120 °C

**Table 2:** Variables used in the manufacturing of hexahedron and monolayer samples.

Property	Extrusion Temperature (°C)	Relative Humidity (%)	Pigmentation
Hexahedral	180, 190, 200, 220, 240 °C	-	Pink, Green, Grey, Transparent
Monolayer	200, 220, 240 °C	16, 50, 98%	Pink, Green, Grey, Transparent

**Table 3:** Influence of storage conditions and operating temperature on the surface finish.

	200 °C	220 °C	240 °C
16%	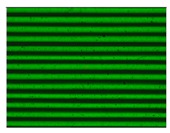	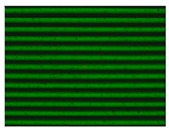	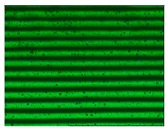
50%	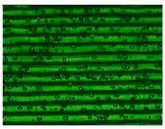	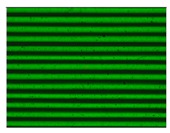	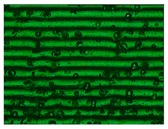
98%	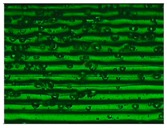	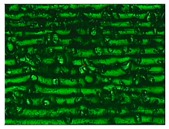	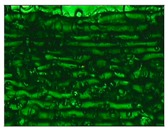

**Table 4:** Elongation of the samples according to their storage conditions.

	16%	50%	98%
**Transparent**	2.215	3.295	2.015
**Pink**	2.312	2.478	2.186
**Grey**	2.417	2.588	1.972
**Green**	2.417	2.200	1.760

**Table 5:** SEM section of monolayer specimens subjected to tensile tests.

16%	50%	98%